# Nutrition and Food Security for All: A Step Towards the Future

**DOI:** 10.3390/nu17071241

**Published:** 2025-04-02

**Authors:** António Raposo, Ariana Saraiva

**Affiliations:** 1 CBIOS (Research Center for Biosciences and Health Technologies), Universidade Lusófona de Humanidades e Tecnologias, Campo Grande 376, 1749-024 Lisboa, Portugal; 2 Research in Veterinary Medicine (I-MVET), Faculty of Veterinary Medicine, Lisbon University Centre, Lusófona University, Campo Grande 376, 1749-024 Lisboa, Portugal; ariana.saraiva@ulusofona.pt; 3 Veterinary and Animal Research Centre (CECAV), Faculty of Veterinary Medicine, Lisbon University Centre, Lusófona University, Campo Grande 376, 1749-024 Lisboa, Portugal

Ensuring food and nutritional security has been a top concern on the global development agenda. Even so, about 600 million people are projected to suffer from chronic undernourishment by 2030 [1]. Because the associated difficulties and growth goals have evolved over time, the necessary action has not been carried out in this field. A more comprehensive agreement that safeguards food security and lowers hunger and malnourishment in all its forms has been approved, in order to support strong economies, healthy populations, and sustainable growth [2,3,4,5,6,7]. Promoting a positive outlook on food, nutrition, and health is essential to eradicate food insecurity in many nations [8]. This may be accomplished by educating individuals about healthy eating choices at a young age through the media, schools, and family. Understanding the importance of a diverse, balanced, and nutrient-rich diet is essential to achieve the second Sustainable Development Goal (SDG) of the global agenda [9]. Furthermore, when creating policies to achieve the SDG 2’s objectives, factors pertaining to security, culture, society, economy, and environment must be taken into account.

When producing food, it is crucial to take into account both the waste generated in the food chain as a result of environmental degradation, and the food waste that could be otherwise consumed by members of society. Finding strategies to boost local and regional food production and consumption is also crucial for promoting sustainable availability, food security, and healthy diets [10]. Other objectives include lowering animal illness rates and producing enough high-quality food in adequate amounts with little waste.

Taking into account these ideas, this Special Issue presents 17 papers published by researchers from 16 different countries all over the world, including Brazil, Chile, China, Greece, Hungary, Iceland, Pakistan, Poland, Portugal, Saudi Arabia, Spain, Thailand, Turkey, the UK, Uruguay, and the USA.

Regarding the review articles included in this Special Issue, it is possible to find one work (scoping review) that addresses the following theme: vitamin B12 supplementation in the vegan population [11]. Because vegans consume less animal products, the researchers came to the conclusion that vitamin B12 insufficiency is prevalent among vegans.

In terms of research articles, we mention 16 relevant works that focus on different areas common to the objectives of this Special Issue: the research conducted by Duda [12] to understand the health-related motivations for urban food self-production in light of semantic field analysis, where the results showed that the inadequate availability of healthy vegetables to residents makes their health concerns a major determinant of their willingness to take part in urban food self-production initiatives; the investigations on urban–rural disparities in food insecurity and weight status among children in the USA, which emphasize the significance of integrating food security interventions into future obesity prevention programs, given the association between food insecurity and increased obesity rates, especially among urban children [13]; and Ahmed et al. [14] published a study on the political economy of maternal child malnutrition: experiences about water, food, and nutrition policies in Pakistan. Nutrition programs encounter challenges including underfunding, weak monitoring, the exclusion of those with low social and cultural capital, corruption in the health system, and the influence of formula milk businesses that are affiliated with the medical community and bureaucracy. In order to increase nutrition security in Pakistan, this study suggests that tackling the macropolitical and economic factors that contribute to undernutrition should be given top priority. Harnirattisai et al. [15] investigated nutritional health risks (food security) in older Thai adults and the related factors, and found that, in Thailand, elderly persons seem to be at a greater risk of malnutrition, especially those with underlying medical conditions and poor incomes. Moreover, using Household Dietary Diversity Scores and spatial analysis to inform food governance in Chile was the topic of the work conducted by del Valle, M. et al. [16]. They stressed how crucial it is to use data insights such as the Household Dietary Diversity Score and spatial visualization to improve food security and guide food governance strategies. Furthermore, a case study in the Federal District of Brazil about food neophobia in children revealed that children from the Federal District exhibit higher levels of neophobia than Brazilian children overall, underscoring the need for more research on the factors that contribute to food phobia in this demographic and nutritional education initiatives to lessen food phobias in the children from this region [17], whilst a qualitative study on healthy food priority areas, barriers, and enablers for equitable healthy food access in Baltimore restaurants was carried out [18]. This study highlights that policies and interventions should demonstrate cultural awareness, give financial support, and provide more precise instructions to assist these companies in overcoming obstacles and gaining access to the resources required for a fair, wholesome food environment. In the works developed by Rozmiarek on the role of nutrition in maintaining the health and physical condition of sports volunteers [19], where it emerged that a large number of volunteers in sports are unaware of how their diet affects their health and fitness, the author pointed out that nutritional education is required to help this population better understand the value of a balanced diet in the context of increasing physical activity. Better nutritional conditions for sporting events should also be provided, and professional sources of knowledge on healthy eating should be encouraged, as well as on the nutritional education needs and preferences of sports volunteers, including access, expectations, and forms of support [20]. Although the volunteers indicated a desire for better nutritional education, more research is required to verify the connection between this knowledge and possible improvements in their health and performance. In another work on the adaptation of the food literacy (FOODLIT) tool for Turkish adults: a validity and reliability study [21], the findings demonstrated the validity and reliability of this adaption. The authors believe that this will be a good tool to measure the level of food literacy among people living in Turkey, and to find out how food literacy affects culinary skills, food production and quality, planning and selection, environmental safety, and food origin. Moreover, the investigation carried out by Ferreira et al. [22] on defatted flaxseed flour as a new ingredient for foodstuffs is a comparative analysis with whole flaxseeds and an updated composition of cold-pressed oil. In line with consumer preferences for natural, low-fat meals, the flour’s importance in promoting food security, the circular economy, and sustainability goals was highlighted by explorations into its role as a minimally processed food ingredient. An analysis from the perspective of sustainability on Mediterranean food pattern adherence in a female-dominated sample of health and social sciences university students [23] highlighted the need for focused initiatives meant to encourage university students to follow Mediterranean food patterns, since this might lead to better health outcomes and more environmental sustainability. Another key piece of research was that conducted by Hernández-Moreno et al. [24] on food security in the rural Mapuche elderly; their analysis and proposals concluded that although rural Mapuche elders continue to follow important practices for their food security, there are major obstacles due to weak policies, migration, and environmental deterioration, while a multi-elemental analysis of energy drinks regarding the nutritional risks of heavy metals in the human diet demonstrated that there may be carcinogenic risks connected to the hazardous element content of energy drinks, in addition to health effects based on their caffeine level. Significant variations in trace element levels between different energy drink products, which may have significant effects on the health and well-being of consumers [25], are also highlighted in this study. Another important study was the comparative analysis of dietary behaviors in children and parents during COVID-19 lockdowns in Greece: insights from a non-representative sample [26]. According to the COVEAT study’s findings, the dietary habits of children and adolescents, as well as those of their parents, were affected differently by each lockdown period. Less positive changes were seen in the second COVID-19 lockdown, which raises the possibility that the introduction of more lockdowns may have had a detrimental effect on people’s lifestyles. Finally, insights from the FINESCOP project on dietary intakes among university students in Iceland were of crucial significance [27]. In this study, Icelandic university students’ food intakes and the relationships between dietary components were investigated. To further comprehend the eating practices of Icelandic university students and investigate the possibility of drawing causal conclusions, more research is required.

Due to the added value provided by all these works, the editors believe that this Special Issue can contribute to deepening the knowledge on nutrition and food security from a global perspective, both for the scientific community and for populations all over the world, and that great steps can be made towards a better future in this context.
